# Standardization of double blind placebo controlled food challenge with soy within a multicentre trial

**DOI:** 10.1186/s13601-016-0129-4

**Published:** 2016-11-07

**Authors:** R. Treudler, A. Franke, A. Schmiedeknecht, B. K. Ballmer-Weber, M. Worm, T. Werfel, U. Jappe, T. Biedermann, J. Schmitt, R. Brehler, A. Kleinheinz, J. Kleine-Tebbe, H. Brüning, F. Ruëff, J. Ring, J. Saloga, K. Schäkel, T. Holzhauser, St. Vieths, J. C. Simon

**Affiliations:** 1 Department of Dermatology, Venerology and Allergology, Universität Leipzig, Leipzig, Germany; 2 Clinical Trial Centre Leipzig (ZKS), Universität Leipzig, Leipzig, Germany; 3 Allergy Unit, Department of Dermatology, University Hospital Zürich, Zurich, Switzerland; 4 Allergy Center Charité, Department of Dermatology, Venerology and Allergology, Charité - Universitätsmedizin Berlin, Berlin, Germany; 5 Department of Dermatology and Allergology, MH Hannover, Hannover, Germany; 6 Division of Clinical and Molecular Allergology Research Center Borstel, Airway Research Center North (ARCN), Borstel, Germany; 7 Department of Internal Medicine, University of Lübeck, Lübeck, Germany; 8 Department of Dermatology, Universität Tübingen, Tübingen, Germany; 9 Department of Dermatology and Allergology, Technical University Munich, Munich, Germany; 10 Department of Dermatology, Medical Faculty Carl Gustav Carus, TU Dresden, Dresden, Germany; 11 Center for Evidence-Based Healthcare, Medical Faculty Carl Gustav Carus, TU Dresden, Dresden, Germany; 12 Department of Dermatology, Universität Münster, Münster, Germany; 13 Department of Dermatology, Elbekliniken Buxtehude, Buxtehude, Germany; 14 Allergy- and Asthma Centre Westend, Berlin, Germany; 15 Day Care Clinic for Allergy and Dermatology, Kiel, Germany; 16 Department of Dermatology and Allergology, Ludwig-Maximilian University, Munich, Germany; 17 Department of Dermatology, University Medical Center, Johannes Gutenberg-University, Mainz, Germany; 18 Department of Dermatology, Ruprecht-Karls-Universität Heidelberg, Heidelberg, Germany; 19 Division of Allergology, Paul-Ehrlich-Institut, Langen, Germany; 20 Leipziger Interdisziplinäres Centrum für Allergologie (LICA) – Comprehensive Allergy Centre (CAC), Klinik für Dermatologie, Venerologie und Allergologie, Universitätsklinikum Leipzig, Philipp-Rosenthal-Straße 23, 04103 Leipzig, Germany

**Keywords:** Birch, Soy, Food allergy, DBPCFC, LOAEL, Methods

## Abstract

**Background:**

Multicentre trials investigating food allergies by double blind placebo controlled food challenges (DBPCFC) need standardized procedures, challenge meals and evaluation criteria. We aimed at developing a standardized approach for identifying patients with birch related soy allergy by means of DBPCFC to soy, including determination of threshold levels, in a multicentre setting.

**Methods:**

Microbiologically stable soy challenge meals were composed of protein isolate with consistent Gly m 4 levels. Patients sensitized to main birch allergen Bet v 1 and concomitant sensitization to its soy homologue Gly m 4 underwent DBPCFC. Outcome was defined according to presence and/or absence of ten objective signs and intensity of eight subjective symptoms as measured by visual analogue scale (VAS).

**Results:**

138 adult subjects (63.8% female, mean age 38 years) underwent DBPCFC. Challenge meals and defined evaluation criteria showed good applicability in all centres involved. 45.7% presented with objective signs and 65.2% with subjective symptoms at soy challenge. Placebo challenge meals elicited non-cardiovascular objective signs in 11.6%. In 82 (59.4%) subjects DBPCFC was judged as positive. 70.7% of DPBCFC+ showed objective signs and 85.4% subjective symptoms at soy challenge. Subjective symptoms to soy challenge meal in DBPCFC+ subjects started at significantly lower dose levels than objective signs (p < 0.001). Median cumulative eliciting doses for first objective signs in DBPCFC+ subjects were 4.7 g [0.7–24.7] and 0.7 g [0.2–4.7] total soy protein for first subjective symptoms (p = 0.01).

**Conclusions:**

We present the hitherto largest group of adults with Bet v 1 and Gly m 4 sensitization being investigated by DBPCFC. In this type of food allergy evaluation of DBPCFC outcome should not only include monitoring of objective signs but also scoring of subjective symptoms. Our data may contribute to standardize DBPCFC in pollen-related food allergy in multicentre settings.

***Trial registration*:**

EudraCT: 2009-011737-27.

## Background

There is still debate whether allergen-specific immunotherapy (AIT) with birch pollen improves birch pollen-related food allergy [1, 2]. One reason for partly contradictory data of previous trials on this topic is the lack of standardized tests to assess clinical reactions to birch pollen-related foods [1, 2]. Double blind placebo controlled food challenge (DBPCFC) is considered as gold standard in diagnosis of food allergy [3]. When performing DBPCFC within multicentre trials, there is a need for standardization of challenge meals (CM), procedures and evaluation criteria [4–8]. In 2012, the PRACTALL group published a proposal for DBPCFC standardization [7], but interpretation of results still mainly depends on the investigator’s judgment [9]. The definition of relevant and comprehensive cut off values for subjective [10, 11] as well as for objective signs is challenging and differences between observers in interpreting DBPCFCs have been shown [12]. Another major problem is the interpretation of symptoms to placebo meals [10]. There exist only few published data on the frequency and type of those placebo reactions [13–15].

With regard to growing numbers of patients with birch pollen-related soy allergy, partly with severe reactions [16–19], we aimed at identifying subjects with birch pollen-related soy allergy by DBPCFC. Here we present a standardized approach for DBPCFC with newly developed challenge meals and evaluation scores that were set up for a multicentre trial. In contrast to challenge meals having been applied in other trials on birch pollen-related food allergies, i.e. to apple or hazelnut [20, 21], we expected best possible standardization of challenge meals due to the availability of purified soy proteins [22]. In a follow-up investigation patients with birch pollen-related soy allergy were to be included in a trial investigating any effect of birch pollen AIT on this type of food allergy [1].

## Methods

### Setting and patient selection

Between January 2010 and February 2013, birch allergic adults (18–65 years) were recruited in 16 centres (15 German, one Swiss). They underwent standardized allergy interview, skin prick test (SPT) with a panel of frequent respiratory allergens including birch (Allergopharma GmbH & Co KG, Reinbek, Germany) and serum IgE test for Bet v 1 and Gly m 4 (ThermoFisher, Freiburg, Germany). Eligibility for DBPCFC was defined at presence of ≥3.5 kU/l of specific IgE for birch allergen Bet v 1 and ≥0.7 kU/l for its soy homologue Gly m 4. Pre-challenge assessments ensured no interference of acute or chronic diseases or drugs. DBPCFC was preferentially performed in the morning to exclude as far as possible confounding factors as heavy meals (a light, allergen free breakfast was allowed) as well as physical or psychological stress. The trial (EudraCT: 2009-011737-27) was approved by the central ethical committee at Universität Leipzig, Germany (No. 230-09-ff-09112009), the local German boards and the local ethical committee Zurich (KEK-2010-0039). All patients gave written informed consent.

### Challenge meal and DBPCFC

Soy and placebo CM consisted of soy free cocoa, carob flour, oat flakes, rice flour, and sugar. Nine doses of isolated soy powder Supro^®^760IP (88% protein, 12% ash, moisture, fat; Uelzena, Uelzen, Germany) were included in soy-containing CM. Single dose levels of soy protein were 0.0004–0.0044–0.05–0.15–0.5–1.5–2.5–5–15 g; resulting in a maximum cumulative dose 24.7 g protein (28.1 g powder). Placebo-CM also contained Sinlac^®^ (rice flour, carob flour, saccharose; Nestlé Nutrition GmbH, Frankfurt, Germany). Five volunteers without known type 1 sensitization to birch or soy participated in triangle test [4] to investigate any sensory differences between soy and placebo meals. For further validation of CM, patients were asked to guess wether they had received active or placebo meal at the last dose level applied. As patients with clinical reactions rather would suspect to have had active CM, we only considered those patients with negative outcome of DBPCFC. Trial sites prepared meals freshly by adding defined amounts of tap water to centrally produced powdered meals (Pharmacy of the University Hospital, Leipzig, Germany). At weeks 0, 6 and 12, all batches of soy meals (levels 4/9), and at week 0, all batches of placebo meals (level 9) and of soy protein isolate were analysed for Gly m 4 contents [22] as well as for microbiological contaminations (Institute of Laboratory medicine, Clinical chemistry and Molecular diagnostics, University Hospital, Leipzig, Germany). DBPCFC was performed on two separate days outside of birch pollen season (randomized sequence of soy and placebo meals). Meals were kept for several seconds in the oral cavity before swallowing. Increasing dose levels were applied at 20 min intervals.

### Evaluation of signs and symptoms

At two meetings, investigators were trained for documentation of ten objective (O) signs (O1-intraoral swelling/blistering, O2-flush, O3-urticaria, O4-angioedema, O5-conjunctivitis, O6-rhinitis, O7-peak flow reduction (PEFR) > 20%, O8-drop of blood pressure (BPD) > 20 mmHg, O9-increase of heart rate (HRI) > 20%, O10-diarrhea/vomiting). Patients recorded subjective (S) symptoms (S1—enoral tingling/itching, S2—perceived lip swelling, S3—itching skin/eye/nose, S4—dysphagia, S5—dyspnea, S6—nausea, S7—abdominal pain, S8—dizziness) on 10 cm visual analogue scales (VAS) [23, 24]. Scales were anchored by the terms “not present”/very strong” at the left/right end of the scale meaning higher scores indicate greater symptom intensity. They were provided with descriptions of subjective symptoms as follows (original in German): S1—I have tingling or scratching or a furry feeling in my throat, S2—My mouth and/or my lips feel big and swollen, S3—I feel itching/tingling/scratching (skin, eyes, nose, ears, hands, feet), S4 I feel constriction, pressure, swelling or lump in my throat, S5—I find it difficult to breathe deeply, S6—I feel sick/nauseated, S7—I feel bloated, heartburn, stomach ache, stomach pain. I have the gripes, S8—I feel dizzy.

Fully blinded investigators followed calculation/classification procedures and assessments of reactions independently from application of soy or placebo CM.

DBPCFC had to be stopped (i) at dose level 9, (ii) at occurrence of definite objective signs or (iii) on patient’s demand. Lowest observed adverse effect level (LOAEL), and median cumulative threshold dose (MCTD) were determined in the study population with positive DBPCFC. A positive reaction to soy or placebo meal was defined if a patient presented at least one of the following three reaction types:

- Objective type—an objective sign as defined (01–10) and/or
- Single subjective type—at least a single subjective symptom as defined (S1–S8) with VAS value reaching 1.5 cm or more and/or
- Subjective sum type—two or more subjective symptoms (S1–S8) with single VAS values each reaching 0.5 cm or more and summing up to 4 cm or more in total.

**Fig. 1:**
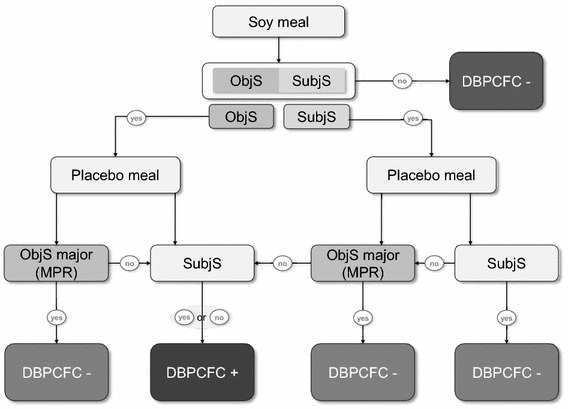
Flow chart for determination of DBPCFC outcome. +, positive; −, negative; ObjS, objective signs; SubjS, subjective symptoms. For definition of major placebo reaction see Table 1

Any objective sign or subjective symptom occurring at placebo meal was classified as either minor or major. Only major placebo reactions (MPR) were taken into account when defining outcome of DBPCFC as positive (+) or negative (−) (including patients with major placebo reactions, Fig. 1). Criteria of MPR were fulfilled, when the intensity of objective signs or subjective symptoms to placebo exceeded those to soy meal (Table 1).

**Table 1:** Definitions of major placebo reactions (MPR) at DBPCFC with examples

Type of major placebo reaction (MPR)	Definition according to signs or symptoms elicited by placebo challenge meal	Example for reaction elicited by placebo challenge meal Dose level/VAS_placebo_	Example for reaction elicited by Soy challenge meal Dose level/VAS
Objective	Any ObjS (O1-10, attributable to the DBPCFC)	Any	Any or none
	Any ObjS (O1-10, not clear if being attributable to the DBPCFC i.e. single wheal) at a lower dose level compared to soy	5 or below	6
Subjective	Any single SubjS* (S1–8) at lower dose level compared to soy	5 or below/any VAS_placebo_ *	6/any VAS_soy_ *
Single*	Any single SubjS* (S1–8) at same dose level like any single SubjS* to soy	5/any VAS_placebo_ *	5/any VAS_soy_ *
Single**	Any single SubjS** (S1–8) at same dose level like any single SubjS to soy and no difference of at least 2 cm in favor of soy	4/any VAS_placebo_ ** (e.g. 0.6 cm)	4/any VAS_soy_ *** < VAS*** _placebo_ -2 cm (e.g. 2 cm)
Sum type***	Two or more aggregated SubjS** (S1–10) and two or more aggregated SubjS** (S1–8) to soy (any but same dose level for both) and no difference between sum VAS values of at least 2 cm in favor of soy	5/any sum VAS_placebo_ *** (e.g. 4 cm)	5/any sum VAS_soy_ *** < sum VAS_placebo_ ***-2 cm (e.g. 5 cm)

*ObjS* objective signs, *SubjS* subjective symptoms, *VAS*_*soy*_ */VAS*_*placebo*_ visual analogue scale value at soy/placebo challenge in cm

* Single VAS of at least 1.5 cm

** Single VAS of at least 0.5 cm

*** Sum of VAS values of at least 0.5 cm, e.g.—example given

### Serology

All subjects were investigated for total IgE and sIgE against Bet v 1 and Gly m 4 (ThermoFisher, Freiburg, Germany) before DBPCFC. 56/82 DBPCFC+ patients being eligible for AIT were further investigated for sIgE against Bet v 2, Gly m 5 and Gly m 6 (ThermoFisher).

### Statistics

Between-group comparisons of DBPCFC+ and DBPCFC− patients were performed descriptively by exact Fisher’s test and Mann–Whitney U test depending on the scale and distribution (binary or ordinal/skewed) of DBPCFC characteristics after soy challenge. In DBPCFC+ patients, symptom characteristics after soy and placebo challenge were compared by McNemar test and Wilcoxon paired rang sum tests. P-values < 0.05 were considered as statistically significant. No adjustment for multiple tests was performed since the aspects investigated primarily served as additional data description.

## Results

### Challenge meals

Triangle tests revealed no significant perceivable differences between soy and placebo CM.

56 patients with negative DBPCFC gave their assumption whether they had received soy or placebo CM. At soy CM (data available for n = 48), 14/48 (29% [95% confidence interval/CI:18; 43]) correctly suspected soy and 34/48 (71% [57; 82]) falsely suspected placebo CM. At placebo CM (data available for n = 45), 18/45 (60% [45; 73]) falsely suspected soy and 27/45 (73% [27; 55]) correctly suspected placebo CM. Overlapping CI between false as well as between correct guesses for placebo and soy indicated no significant differences between both CM.

Quantification of Gly m 4 levels (aggregated over all batches) gave

- 0.012610% (CI 0.011741; 0.013478%.) in pure soy Supro^®^760IP;
- 0.000410% (CI 0.000386; 0.000434) in dose level 4 and
- 0.002164% (CI 0.002051; 0.002278) in dose level 9.

Coefficients of variation at or below 20% were found for the predefined shelf life of 12 weeks in all series measured. No Gly m 4 was-detectable in any placebo batch with respect to the detection limit of the ELISA used [22]. No significant microbiological impurities were detected in any batch within these 12 weeks. Preparation and application of CM was easy to handle in all centres.

### Study population

195 patients (63.6% female, mean (standard deviation) 38.1 (12.8) years) were screened, 193 (95.6%) had positive SPT to birch. 80 (41%) reported on previous symptoms upon consumption of soy containing foods, which were mostly of mild character. 138 (63.8% female, 38 (12.6) years) underwent DBPCFC (276 challenges). 82 (59.4%) fulfilled the criteria of positive DBPCFC (62.2% female, 37.0 (13.6) years. 81 (98.8%) DBPCFC+ subjects had allergic rhinitis, 41 (50%) allergic asthma, 26 (31.7%) atopic eczema.

### Evaluation of DBPCFC

Maximum dose 9 was applied in 104/138 (75.4% [95%-CI: 67.6; 81.8]) at soy and in 125 (90.6 [84.5; 94.4] %) at placebo challenge (significant differences). Objective signs occurred in 63 (45.6 [38.3; 54.7] %) at soy as well as in 30 (21.7 [13.8; 27.0] %) at placebo challenge (statistically significant). Median lowest dose [IQR] which induced objective signs was dose 6 [4–7] at soy and 5 [3–7] at placebo challenge.

**Fig. 2:**
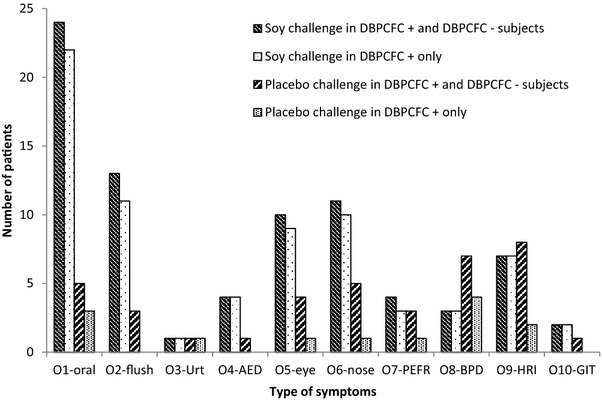
Objective signs at lowest objective sign dose to soy and to placebo challenge meals. 138 subjects underwent DBPCFC, 82/138 were DBPCFC +. At soy challenge, 51/138 (24/82 DBPCFC+) patients had no objective signs and 9/82 DBPCFC+ presented with more than one sign. At placebo challenge, 71/138 (71/82 DBPCFC+) had no objective signs. Urt, urticaria; AED, angioedema; PEFR, peak flow reduction > 20%; BPD, drop of blood pressure > 20 mmHg; HRI, heart rate increase > 20%; GIT, gastrointestinal symptoms

**Fig. 3:**
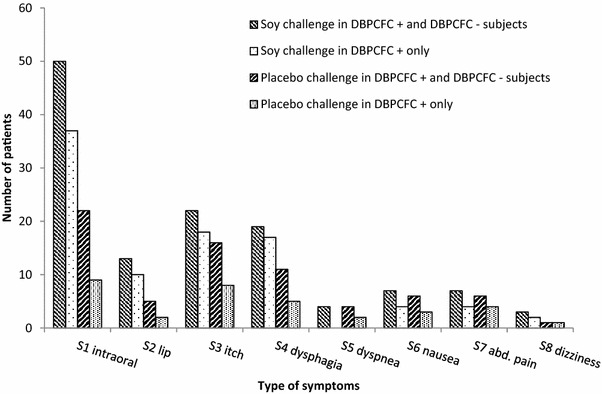
Subjective symptoms at lowest subjective symptom dose to soy and to placebo challenge meals. 138 subjects underwent DBPCFC, 82/138 wereDBPCFC+. Intraoral, tingling/itching; lip, swelling; itch, skin/eye/nose; abd., abdominal

Subjective symptoms occurred in 90 (65.2 [95%-CI: 57.0; 72.7] %) at soy as well as in 57 (41.3 [33.4; 49.6] %) at placebo challenge (statistically significant). Single subjective type symptoms were observed in all patients with subjective reactions while sum type subjective symptoms occurred in 72 (52.2 [43.9; 60.3] %) to soy and 46 (33.3 [26.0; 41.6] %) to placebo (statistically significant). Median lowest dose [IQR] which induced subjective symptoms was dose 5 for both soy and placebo [3–6 and 2–7; respectively]. All objective signs and subjective symptoms were predominantly of the mucocutaneous type (Figs. 2 and 3).

In 82/138 (59%) subjects, DBPCFC was judged as positive. Detailed data on objective signs and subjective symptoms in DBPCFC+ and DBPCFC− subjects as well as numbers of major placebo reactions are given in Table 2. Subjective placebo reactions (single and/or sum values) were reported by 41% (n = 57). Characteristics of objective signs and subjective symptoms of DBPCFC+ and DBPCFC− subjects are given in Fig. 2 and 3. Significant differences between DBPCFC+ and DBPCFC− patients were demonstrated with regard to occurrence of objective signs and subjective symptoms (Table 2).

**Table 2:** Characteristics of DBPCFC positive and DBPCFC negative subjects

	DBPCFC negative (n)	DBPCFC positive (n)	differences with α = 5% between groups
Number of subjects	56 (40.6%)	82 (59.4%)	
Maximum soy challenge meal level 9 applied	53 (94.6% [85.4; 98.2])	51 (62.2% [51.4; 71.9])	Significant
Maximum placebo challenge meal level 9 applied	48 (85.7%[74.3; 92.6])	77 (93.9% [86.5; 97.4])	n.s.
Objective signs (O1-10) at soy challenge meal (in n patients)	5 (8.9% [3.9; 19.3])	58 (70.7% [61.4; 80.5])	Significant
Subjective symptoms (S1–8) at soy challenge meal (single type or sum type)	20 (35.7% [24.5; 48.8])	70 (85.4% [76.1; 91.4])	Significant
Major placebo reactions objective type^a^	19 (33.9% [24.5; 48.8])	0 (0% [0; 4.5])	Significant
Major placebo reactions subjective type^a^	27 (48.2% [35.7; 61.0])	10 (12.2% [6.8; 21.0])	Significant

Maximum dose levels applied and occurrence of objective signs, subjective symptoms and major placebo reactions in DBPCFC positive and DBPCFC negative patients at soy and placebo challenge meals. 95% confidence intervals are given (per cent based on the number of patients per group; in case of non-overlapping confidence intervals significant differences with α = 5% between populations exist)

*n.s.* not significant

^a^For definition of Major placebo reactions see Table 1

#### Characteristics of patients with positive DBPCFC

58/82 DBPCFC+ subjects (70.7 [95%-CI: 61; 80]%) showed objective signs and 70 (85.4.4 [76.1; 91.4]%) subjective symptoms at soy challenge (Figs. 2, 3). 11 (13.4. [7.7; 22.4]%) DBPCFC+ subjects had objective signs and 26 (31.7 [22.6; 42.4]%) subjective symptoms at placebo challenge. The majority of objective signs caused by soy CM were of the mucocutaneous type (Fig. 2). No objective signs occurred in 24 (29.2%) subjects at soy CM and in 71 (86.6%) at placebo CM.

Ten (12.2%) of DBPCFC+ subjects patients reported MPR of subjective type to placebo (Table 2) but all of them had more extended objective signs to soy.

In DBPCFC+ patients, median single/maximum VAS values [IQR] at lowest dose of occurrence were 2.2 cm [1.6–3.3] at soy and 0 cm [0–1.6] at placebo challenge. In contrast, in DBPCFC− subjects, VAS values were 0 cm [0–1.7] at soy and 1.5 cm [0–2.3] at placebo challenge.

Subjective symptoms at soy challenge started mostly at lower dose levels (median dose 5 [IQR 3–6]) than objective signs (median dose 6; [4–7]) (p = 0.01; Fig. 3).

**Fig. 4:**
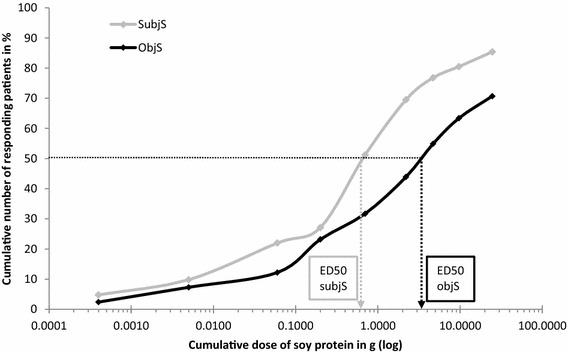
Cumulative dose levels of soy protein at occurrence of first signs and symptoms. Objective signs (ObjS) occurred in 58/82 (70.7%) and subjective symptoms (SubjS, either single or sum type) in 70/82 (85.4%) DBPCFC+ patients, respectively. SubjS to soy challenge meal started at significantly lower dose levels than ObjS (p < 0.001). Cumulative threshold dose eliciting objS and subjS in 50% of subjects (ED_50_) can be extrapolated

MCTD [IQR] for first objective signs was 4.7 g [0.7–24.7] and for first subjective symptoms 0.7 g (0.2–4.7) soy protein (p = 0.01). Cumulative threshold doses of soy protein eliciting objective signs or subjective symptoms in 50% of subjects (ED50) can be extrapolated from Fig. 4. Four DBPCFC+ patients showed subjective symptoms and two objective signs at first dose level which therefore corresponded to LOAELs.

#### Serology

In 138 patients with DBPCFC, total IgE (median [IQR]) was 165 kU/l [87.4–417], sIgE against Bet v 1 34 kU/l [17.3–68.7] and against Gly m 4 8.3 kU/l [0.8–3.8]. In DBPCFC+, sIgE against Bet v 1 was 34 kU/l [17.3–68.7] versus 35.6 kU/l [16.9–60] in DBPCFC− and against Gly m 4 8.3 kU/l [3.8–16.2] vs. 5.67 kU/l [2.8–17.03], respectively (n.s.). Further sIgE in 56/82 DBPCFC+, randomized for AIT, was positive against Bet v 2 in 9/56 and low level sIgE was found against both Gly m 5 and Gly m 5 in only 1/56 patients.

## Discussion

Here we present the hitherto largest group of adults with combined sensitization to birch allergen Bet v 1 and to its soy homologue Gly m 4 being investigated by DBPCFC. In this multicentre trial, 82 subjects (59.4%) had positive DBPCFC according to harmonized evaluation criteria. With the aim of identifying DBPCFC positive patients being eligible for a follow-up trial on AIT against birch pollen allergen, we decided to only include patients with defined IgE cut off values, though being aware that IgE values cannot safely predict reactivity at DBPCFC [25]. As clinical symptoms may occur in Bet v 1 sensitized subjects already at first consumption of soy products [26], our inclusion criteria did not ask for history of clinical allergy. Since no recipe was available at the planning stage of our trial, newly developed challenge meals were composed as microbiologically stable desserts based on powdered ingredients. Soy protein levels of active meals were chosen on the basis of previously published data [27].

Details on DBPCFC evaluation criteria used in clinical trials are rarely reported in the current literature and no consensus exists on how to discriminate positive from negative outcomes. Presentation of detailed evaluation criteria used in this trial in comparison with criteria of PRACTALL [8] and EuroPrevall [27] is given in Table 3.

**Table 3:** Comparison of different DBPCFC evaluation criteria

	EuroPrevall [28; from 2015]	PRACTALL [8, from 2012]	This trial (2008)
Scoring	Objective signs absent/present Subjective symptoms: persistent > 45 min	Objective signs and subjective symptoms 0 = absent, 1 = mild, 2 = moderate, 3 = severe	Objective signs: present/absent Subjective symptoms: VAS (0–10 cm)
Skin/mucosa	Blisters of oral mucosa (O) Skin flushing (O) Urticaria (O) Angioedema (O) Conjunctivitis (O) Itching of palms, soles head (S)	Pruritus (S) Urticaria/angioedema (O) Rash (O)	O1 Intraoral swelling/blistering O2 Flush O3 Urticaria O4 Angioedema O5 Conjunctivitis S1 Intraoral tingling/itching S2 Perceived lip swelling S3 Itching skin/eye/nose
Upper respiratory tract	Rhinitis (O)	Sneezing/itching (S/O)	O6 Rhinitis S3 Itching nose
Lower respiratory tract	Drop of FEV1 > 12% or drop of PEF > 20% (O) Laryngeal edema (O)	Wheezing (O) Laryngeal (O)	O7 Peak flow reduction > 20% S5 Dyspnea
Gastrointestinal tract	Diarrhea (O) Emesis (S) Severe gastric/abdominal pain (S)	Itchy mouth/throat, nausea, pain (S) emesis, diarrhea (O)	O10 Diarrhea, vomiting S4 Dysphagia S6 Nausea S7 Abdominal pain
Cardiovascular/neurological system	Drop of BP > 20 mmHg (O)	Heart rate increase or drop in blood pressure, dizziness, unconsciousness (O/S)	O8 Drop of BP > 20 mmHg O9 Increase of heart rate > 20% S8 Dizziness

*S* subjective, *O* objective, *BP* blood pressure

In our DBPCFC+ patients, objective signs were most frequently of the mucocutaneous type. At placebo challenge, fifty percent (15/30) of all objective signs were due to heart rate increase and drop of blood pressure. Both may be symptoms of immediate type reaction but may as well be induced by psychovegetative factors. Thus, as a single parameter, neither of these cardiovascular symptoms seemed appropriate for identifying DBPCFC+ patients. Also, flush, rhinitis and conjunctivitis, possibly against the background of underlying atopic diseases, were more frequently seen in patients judged as DBPCFC− than in DBPCFC+ patients at placebo challenge.

Regarding subjective symptoms at DBPCFC, there is no consensus on how to monitor or how to score. In our trial as well as in a recent report [29], visual analogue scales (VAS), were used. We predefined cut off values for data analyses with regard to experiences from pain measurement [23, 24]. As VAS values are prone to a certain risk of being unspecific, we requested a VAS value of 1.5 cm or more to be classified as a single positive reaction. Most of subjective symptoms documented in our trial were typical for contact urticaria of the oropharyngeal sites [17, 18].

For evaluation of pollen-related food allergy with frequent predominance of subjective symptoms [17, 18], our evaluation criteria may be more comprehensive than those proposed by PRACTALL [8] which neither consider in detail mucocutaneous symptoms nor include measuring of symptom’s degree. However, we are aware that the complex definition of major subjective placebo reactions presented for this trial may not be suitable for routine use.

It was recently shown, that standardized food challenges are subject to a great variability in interpretation of clinical symptoms [9]. Regarding placebo events, in the current literature, no systematic documentation in DBPCFC in adults, like in our trial, has been published. In children, objective signs and/or subjective symptoms occurred in at least 2.8% [12] or 12.9% [14] of challenges. We documented an overall number of objective placebo reactions in 21.6% which highlightens the complexity of DBPCFC evaluation.

DBPCFC is usually considered positive when objective signs occur exclusively on active and not on placebo challenge [8, 28, 29]. This definition is insufficient in birch pollen related food allergy were patients often suffer from subjective symptoms only and/or may present objective signs against their atopic background not being induced by DBPCFC. We suggest that minor placebo reactions should be compatible with positive DBPCFC outcome supposed that there are relevant differences between reactions at active and at placebo meal. With the aim of harmonization in a multicentre setting, we therefore suggested a definition of relevant (major) placebo reactions (Table 1).

According to theses definitions, we identified MCTD for subjective symptoms (0.7 g) and for objective signs (4.7 g), which are, however, not considered representative for all subjects with birch related soy allergy due to selected inclusion criteria. Nevertheless, data were close to those determined in a previous trial on soy allergy with MCTD of 0.9 (subjective) and 4.8 g (objective), respectively [27].

Due to our experience we feel that evaluation of DBPCFC outcome, only can be standardized to a certain extend. Final decision upon relevance of occurring sign and symptoms during DBPCFC still relies on the clinical investigator, especially with regard to circumstances during the challenge procedure as well as considering any physical and/or psychological comorbidities of the patient.

## Conclusions

We present a standardized approach for DBPCFC with soy that includes application of challenge meals with stable Gly m 4 values as well as determination of threshold levels in a multicentre setting. For evaluation of any treatment effects on birch pollen-related food allergy, we see an urgent need not only for providing standardized challenge meals but also for investigating validity and reliability of DBPCFC outcome scoring systems taking into account intensity of objective clinical signs as well as subjective symptoms.

## Abbreviations

CI: confidence interval
CM: challenge meal
DBPCFC: double blind placebo controlled food challenge
IQR: interquartile range
LOAEL: lowest observed adverse effect level
MCTD: median cumulative threshold dose
MPR: major placebo reaction
O1-O10: objective signs of different types (1–10)
ObjS: objective signs
S1–S8: subjective symptoms of different types (1–8)
SubjS: subjective symptoms
VAS: visual analogue scale

## References

[1] Treudler R, Simon JC (2012). Severe soy allergy in adults. Is there a role for specific immunotherapy?. Hautarzt.

[2] Kinaciyan T, Nagl B, Faustmann S, Kopp S, Wolkersdorfer M, Bohle B (2016). Recombinant Mal d 1 facilitates sublingual challenge tests of birch pollen-allergic patients with apple allergy. Allergy.

[3] Bindslev-Jensen C, Ballmer-Weber BK, Bengtsson U, Blanco C, Ebner C, Hourihane J, Knulst AC, Moneret-Vautrin DA, Nekam K, Niggemann B, Osterballe M, Ortolani C, Ring J, Schnopp C, Werfel T (2004). Standardization of food challenges in patients with immediate reactions to foods—position paper from the European Academy of Allergology and Clinical Immunology. Allergy.

[4] Cochrane SA, Salt LJ, Wantling E, Rogers A, Coutts J, Ballmer-Weber BK, Fritsche P, Fernández-Rivas M, Reig I, Knulst A, Le TM, Asero R, Beyer K, Golding M, Crevel R, Clare Mills EN, Mackie AR (2012). Development of a standardized low-dose double-blind placebo-controlled challenge vehicle for the EuroPrevall project. Allergy.

[5] Vlieg-Boerstra BJ, Bijleveld CM, van der Heide S, Beusekamp BJ, Wolt-Plompen SA, Kukler J, Brinkman J, Duiverman EJ, Dubois AE (2004). Development and validation of challenge materials for double-blind, placebo-controlled food challenges in children. J Allergy Clin Immunol.

[6] Muraro A, Werfel T, Hoffmann-Sommergruber K, Roberts G, Beyer K, Bindslev-Jensen C, Cardona V, Dubois A, Toit G, Eigenmann P, Fernandez Rivas M, Halken S, Hickstein L, Høst A, Knol E, Lack G, Marchisotto MJ, Niggemann B, Nwaru BI, Papadopoulos NG, Poulsen LK, Santos AF, Skypala I, Schoepfer A, Van Ree R, Venter C, Worm M, Vlieg-Boerstra B, Panesar S, de Silva D, Soares-Weiser K, Sheikh A, Ballmer-Weber BK, Nilsson C, de Jong NW, Akdis CA EAACI Food Allergy and Anaphylaxis Guidelines Group (2004). EAACI Food Allergy and Anaphylaxis Guidelines: diagnosis and management of food allergy. Allergy.

[7] Asero R, Fernandez-Rivas M, Knulst AC, Bruijnzeel-Koomen CA (2009). Double-blind, placebo-controlled food challenge in adults in everyday clinical practice: a reappraisal of their limitations and real indications. Curr Opin Allergy Clin Immunol.

[8] Sampson HA, GerthvanWijk R, Bindslev-Jensen C, Sicherer S, Teuber SS, Burks AW, Dubois AE, Beyer K, Eigenmann PA, Spergel JM, Werfel T, Chinchilli VM (2012). Standardizing double-blind, placebo-controlled oral food challenges: American Academy of Allergy, Asthma & Immunology-European Academy of Allergy and Clinical Immunology PRACTALL consensus report. J Allergy Clin Immunol.

[9] van Erp FC, Knulst AC, Meijer Y, Gabriele C, van der Ent CK (2014). Standardized food challenges are subject to variability in interpretation of clinical symptoms. Clin Transl Allergy.

[10] Gellerstedt M, Magnusson J, Gråjö U, Ahlstedt S, Bengtsson U (2004). Interpretation of subjective symptoms in double-blind placebo-controlled food challenges—interobserver reliability. Allergy.

[11] Agache I, Bilò M, Braunstahl GJ, Delgado L, Demoly P, Eigenmann P, Gevaert P, Gomes E, Hellings P, Horak F, Muraro A, Werfel T, Jutel M (2015). In vivo diagnosis of allergic diseases–allergen provocation tests. Allergy.

[12] Brand PL, Landzaat-Berghuizen MA (2014). Differences between observers in interpreting double-blind placebo-controlled food challenges: a randomized trial. Pediatr Allergy Immunol.

[13] Ahrens B, Niggemann B, Wahn U, Beyer K (2014). Positive reactions to placebo in children undergoing double-blind, placebo-controlled food challenge. Clin Exp Allergy.

[14] Niggemann B, Beyer K (2005). Diagnostic pitfalls in food allergy in children. Allergy.

[15] Vlieg-Boerstra BJ, van der Heide S, Bijleveld CM, Kukler J, Duiverman EJ, Dubois AE (2007). Placebo reactions in double-blind, placebo-controlled food challenges in children. Allergy.

[16] Treudler R, Werner M, Thiery J, Kramer S, Gebhardt C, Averbeck M, Simon JC (2008). High risk of immediate-type reactions to soy drinks in 50 patients with birch pollinosis. J Investig Allergol Clin Immunol.

[17] Beyer S, Franke A, Simon JC, Treudler R (2016). Measurement of health-related quality of life in adult patients with birch pollen associated food allergy. J Deutsch Dermatol Ges.

[18] Werfel T, Asero R, Ballmer-Weber BK, Beyer K, Enrique E, Knulst AC, Mari A, Muraro A, Ollert M, Poulsen LK, Vieths S, Worm M, Hoffmann-Sommergruber K (2015). Position paper of the EAACI: food allergy due to immunological cross-reactions with common inhalant allergens. Allergy.

[19] Mittag D, Vieths S, Vogel L, Becker WM, Rihs HP, Helbling A, Wüthrich B, Ballmer-Weber BK (2004). Soybean allergy in patients allergic to birch pollen: clinical investigation and molecular characterization of allergens. J Allergy Clin Immunol.

[20] Hansen KS, Khinchi MS, Skov PS, Bindslev-Jensen C, Poulsen LK, Malling HJ (2004). Food allergy to apple and specific immunotherapy with birch pollen. Mol Nutr Food Res.

[21] van Hoffen E, Peeters KA, van Neerven RJ, van der Tas CW, Zuidmeer L, van Ieperen-van Dijk AG, Bruijnzeel-Koomen CA, Knol EF, van Ree R, Knulst AC. Effect of birch pollen-specific immunotherapy on birch pollen-related hazelnut allergy. J Allergy Clin Immunol. 2011;127(1):100–1, 101.e1–3. 10.1016/j.jaci.2010.08.021 20933256

[22] Holzhauser T, Franke A, Treudler R, Schmiedeknecht A, Randow S, Becker WM, Lidholm J, Rösch P, Vieths S, Simon JC (2016). The BASALIT multicentre trial: Gly m 4 quantification for consistency control of challenge meal batches and towards Gly m 4 threshold data. Mol Nutr Food Res..

[23] Flynn D, Van Schaik P, Van Wersch A (2004). A comparison of multi-item Likert and Visual Analogue Scales for the assessment of transactionally defined coping function. Eur J Psychol Assess.

[24] Hawker GA, Mian S, Kendzerska T, French M (2011). Measures of adult pain: Visual Analog Scale for Pain (VAS Pain), Numeric Rating Scale for Pain (NRS Pain), McGill Pain Questionnaire (MPQ), Short-Form McGill Pain Questionnaire (SF-MPQ), Chronic Pain Grade Scale (CPGS), Short Form-36 Bodily Pain Scale (SF-36 BPS), and Measure of Intermittent and Constant Osteoarthritis Pain (ICOAP). Arthritis Care Res (Hoboken).

[25] Celik-Bilgili S, Mehl A, Verstege A, Staden U, Nocon M, Beyer K, Niggemann B (2005). The predictive value of specific immunoglobulin E levels in serum for the outcome of oral food challenges. Clin Exp Allergy.

[26] Süss A, Rytter M, Sticherling M, Simon JC (2005). Anaphylactic reaction to soy drink in three patients with birch pollen allergy. J Dtsch Dermatol Ges.

[27] Ballmer-Weber BK, Holzhauser T, Scibilia J, Mittag D, Zisa G, Ortolani C, Poulsen LK, Vieths S, Bindslev-Jensen C (2007). Clinical characteristics of soybean allergy in Europe: a double-blind, placebo-controlled food challenge study. J Allergy Clin Immunol..

[28] Fernández-Rivas M, Barreales L, Mackie AR, Fritsche P, Vázquez-Cortés S, Jedrzejczak-Czechowicz M, Kowalski ML, Clausen M, Gislason D, Sinaniotis A, Kompoti E, Le TM, Knulst AC, Purohit A, de Blay F, Kralimarkova T, Popov T, Asero R, Belohlavkova S, Seneviratne SL, Dubakiene R, Lidholm J, Hoffmann-Sommergruber K, Burney P, Crevel R, Brill M, Fernández-Pérez C, Vieths S, Clare Mills EN, van Ree R, Ballmer-Weber BK (2015). The EuroPrevall outpatient clinic study on food allergy: background and methodology. Allergy.

[29] Gellerstedt M, Bengtsson U, Niggemann B (2007). Methodological issues in the diagnostic work-up of food allergy: a real challenge. J Investig Allergol Clin Immunol.

